# Editorial: Fungal-plant interactions in a changing environment: from mutualism to pathogenesis

**DOI:** 10.3389/ffunb.2026.1852814

**Published:** 2026-04-28

**Authors:** Subhadeep Das, Adreeja Basu, Paola Angelini, Soumyadev Sarkar

**Affiliations:** 1 Department of Biotechnology, School of Life Science & Biotechnology, Adamas University, Kolkata, West Bengal, India; 2 Marxe School of Public and International Affairs, Baruch College, City University of New York, New York, NY, United States; 3 Dipartimento di Chimica, Biologia e Biotecnologie, Università degli Studi di Perugia, Perugia, Italy; 4 Center for Fundamental and Applied Microbiomics, Biodesign Institute, Arizona State University, Tempe, AZ, United States

**Keywords:** abiotic stress, AMF, climate change, fungi, mutualism, pathogenesis, plant, plant-fungal associations

Fungal-plant interactions are key to ecosystem functioning, which influences nutrient cycles, plant growth, and diseases. This wide-spectrum relation ranges from beneficial associations, such as mycorrhizal fungi that help plants absorb nutrients, to harmful fungi that can cause disease. These interactions are often impacted by global environmental changes, including rising temperatures, shifting rainfall patterns, elevated CO_2_ levels, and human activities such as deforestation and unsustainable agriculture. As a result, fungi that once supported plant growth can become detrimental under altered conditions. This transition has profound implications for plant health, biodiversity, and agricultural productivity (**Figure 1**). This is an ever-evolving topic, and addressing these challenges requires a deeper understanding of the mechanisms driving these shifts and harnessing this knowledge to develop more resilient and sustainable agricultural systems. The goal of this Research Topic, therefore, is to investigate how environmental changes restructure fungal-plant interactions across the mutualism-pathogenesis continuum. Drawing on studies of fungal symbiosis, nutrient-dependent interaction shifts, climate-mediated pathogen emergence, and transcriptomic reprogramming of host-fungal relationships, it seeks to identify the key drivers and mechanisms that govern these transitions. The Research Topic aims to attract researchers specializing in the field of the fungal-plant interface.

**Figure 1:**
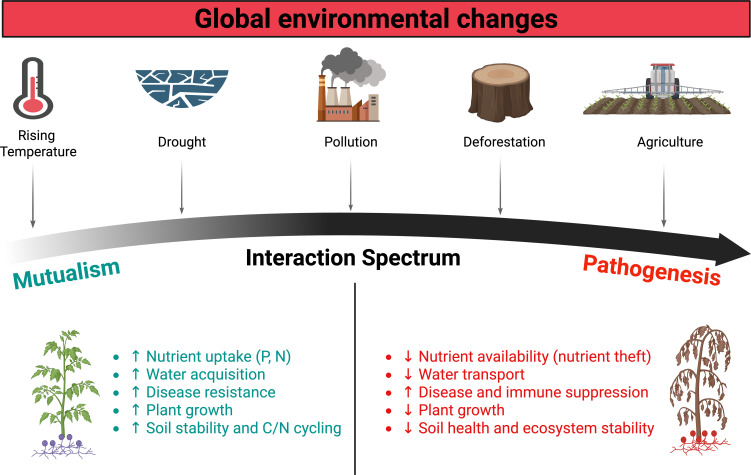
Fungal-plant interactions in a changing environment (Created with BioRender.com).

## The fungal-plant interaction continuum

A comprehensive review by Mishra et al. discusses fungal-plant interactions, focusing on how environmental factors shape the mutualistic and pathogenic relationships. Their work highlights the symbiotic role of mycorrhizal associations in enhancing plant health and ecosystem stability, while also addressing the threats posed by fungal pathogens to agricultural and natural systems. The review emphasizes that the influence of biotic and abiotic drivers, such as temperature, moisture, soil conditions, the presence of other microbes, herbivores, and competing plants, determines the direction of these relationships. It also discusses the broader implications for ecosystem productivity, conservation, and sustainable agriculture, addresses future challenges related to climate change and emerging pathogens, and emphasizes the need for genomic and ecological insights to inform restoration and management strategies.

## Fungal symbiosis improves plant tolerance to drought stress

Changes in precipitation patterns increase the frequency of drought events, disrupting plant systems and agricultural productivity. Das et al. shed light on the positive role of arbuscular mycorrhizal fungi (AMF) symbiosis in enhancing plant tolerance to drought stress. The mini-review focuses on diverse AMF-mediated mechanisms that benefit plants, including antioxidant defenses, phytohormone mediations, osmotic adjustments, proline expression, fungal water absorption and transport, morphological modifications, and photosynthesis. Understanding these mechanisms is key to developing sustainable agricultural practices in anticipation of future drought events.

## Context-dependent transitions between mutualism and antagonism

The study by Sidek et al. illustrates how plant immune signaling and nutrient availability together shape the outcome of fungal-plant interactions. Using *Arabidopsis thaliana* and the root endophyte *Colletotrichum tofieldiae* system, the authors explain context-dependent transitions of these associations. Under phosphate-limited conditions, reciprocal nutrient and carbon exchange supports a mutualistic interaction that is tightly regulated by salicylic acid signaling, which restrains fungal proliferation. In contrast, sufficient phosphate disrupts this balance, diminishing the benefits to the host and shifting the interaction toward competition.

## Climate change and the emergence of disease dynamics

Awais et al. demonstrate that global warming directly alters fungal epidemiology and disease emergence. A shift in snowfall patterns enables cold-tolerant strains of *Puccinia striiformis* f.sp. *tritici* to persist under snow and contributes to earlier-than-expected stripe rust epidemics in wheat. The authors suggest the possibility of invasion by migratory genotypes or continued sporulation occurring after infection beneath the snow.

## Molecular reprogramming facilitates pathogenicity

Another study by Quintero-Mercado et al. uses transcriptomic analysis to show that host gene expression promotes the pathogenicity of an endophyte. Ripe mango fruit cv. Azúcar, upon infection by *Colletotrichum tropicale*, activates mitogen-activated protein kinase signaling, pathogen-associated molecular pattern-triggered immunity, and phenylalanine metabolism. Although mango fruit mounts a heightened defense response against *C. tropicale*, it fails to overcome the pathogen’s initial quiescent phase and instead creates conditions that facilitate its establishment by suppressing oxidative burst pathways, which may later contribute to oxidative stress during the necrotrophic phase.

## Conclusion

These high-quality contributions endorse the idea that fungal-plant interactions are dynamic in nature and shaped by environmental pressures, host physiology, and molecular recognition. Mutualism and antagonism are often context-dependent, and this knowledge can be very useful in the management of the balance between symbiosis and disease in a rapidly evolving world.

